# The GLP-1 agonist, exendin-4, stimulates LH secretion in female sheep

**DOI:** 10.1530/JOE-23-0105

**Published:** 2023-08-17

**Authors:** Elizabeth M Simpson, Iain J Clarke, Christopher J Scott, Cyril P Stephen, Alexandra Rao, Allan J Gunn

**Affiliations:** 1 School of Agricultural, Environmental and Veterinary Sciences, Faculty of Science and Health, Charles Sturt University, Wagga Wagga, NSW, Australia; 2 Gulbali Institute, Charles Sturt University, Wagga Wagga, NSW, Australia; 3 School of Agriculture Food and Ecosystem Sciences, Faculty of Science, The University of Melbourne, Parkville, VIC, Australia; 4 School of Dentistry and Medical Science, Faculty of Science and Health, Charles Sturt University, Wagga Wagga, NSW, Australia

**Keywords:** preproglucagon, exendin-4, sheep, luteinising hormone

## Abstract

Our previous studies showed that microinjection into the median eminence of the sheep of glucagon-like peptide- 1 (GLP-1) or its receptor agonist exendin-4 stimulates luteinising hormone (LH) secretion, but it is unknown whether the same effect may be obtained by systemic administration of the same. The present study measured the response in terms of plasma LH concentrations to intravenous (iv) infusion of exendin-4. A preliminary study showed that infusion of 2 mg exendin-4 into ewes produced a greater LH response in the follicular phase of the oestrous cycle than the luteal phase. Accordingly, the main study monitored plasma LH levels in response to either 0.5 mg or 2 mg exendin-4 or vehicle (normal saline) delivered by jugular infusion for 1 h in the follicular phase of the oestrous cycle. Blood samples were collected at 10 min intervals before, during and after infusion. Both doses of exendin-4 increased mean plasma LH concentrations and increased LH peripheral pulse amplitude. There was no effect on inter-pulse interval or timing of the preovulatory LH surge. These doses of exendin-4 did not alter plasma insulin or glucose concentrations. Quantitative PCR of the gastrointestinal tract samples from a population of ewes confirmed the expression of the preproglucagon gene (*GCG*). Expression increased aborally and was greatest in the rectum. It is concluded that endogenous GLP-1, most likely derived from the hindgut, may act systemically to stimulate LH secretion. The present data suggest that this effect may be obtained with levels of agonist that are lower than those functioning as an incretin.

## Introduction

The level of nutrition and body composition has a significant effect on reproductive function (Clarke & Arbabi 2016). In ruminants, as in other species, energy and nutrient restriction reduces the secretion of gonadotrophin-releasing hormone (GnRH), luteinising hormone (LH) and follicle stimulating hormone (FSH), may delay puberty and delay the return to cyclicity following parturition or lactation. This is associated with reduced conception rates, reduced ovulation rates/ovulation failure and poor-quality oocytes (Robinson 1996, Robinson *et al.* 2006, De Bond & Smith 2014, Dupont *et al.* 2014). Conversely, beef cows in good condition at calving and receiving a high intake diet post-calving have a shortened anoestrous period (Wright *et al.* 1992), whilst nutritional ‘flushing’ of ewes with high-quality feed before mating increases ovulation rate and litter size (Robinson *et al.* 2006).

These effects of nutrition on reproduction are, at least in part, relayed to GnRH neurons and/or gonadotrophs by a range of hormones and neuropeptides. In general, neuropeptides that stimulate food intake have an inhibitory effect on the gonadotrophic axis, whereas those which reduce food intake have a stimulatory effect on the same (Clarke 2014). For example, the orexigenic peptide, neuropeptide Y (NPY), reduces pulsatile LH secretion (Barker-Gibb *et al.* 1995), whereas melanocortins, which are anorexigenic, stimulate pulsatile LH secretion in OVX ewes (Backholer *et al.* 2010). The function of these neuropeptides is regulated by several appetite-regulating hormones. Thus, leptin, which is a fat-derived hormone, reduces appetite but has a positive effect on the gonadotrophic axis, restoring pulsatile LH secretion in undernourished OVX ewes (Henry *et al.* 2004). Conversely, the appetite stimulant ghrelin, which originates predominantly from the abomasum and small intestine in sheep (Hayashida *et al.* 2001, Grouselle *et al.* 2008), reduces pulsatile LH secretion in OVX ewes (Iqbal *et al.* 2006).

Another circulating appetite-regulating peptide is glucagon-like peptide 1 (GLP-1) encoded by the preproglucagon gene (*GCG*) (Huda *et al.* 2006, Sandoval & D'Alessio 2015, Sekar 2017). GLP-1 is an anorexigen secreted from intestinal L-cells in response to eating and the presence of ingesta in the intestinal lumen (Paternoster & Falasca 2018). GLP-1 is also found in neurons of the nucleus of the solitary tract in the hindbrain (Jin *et al.* 1988, Paternoster & Falasca 2018), which project to rostral centres in the brain. With the glucose-dependent insulinotropic polypeptide (GIP), GLP-1 is an important incretin (Baggio & Drucker 2007) and GLP-1 agonists are widely used in the management of type 2 diabetes mellitus (Wang *et al.* 2022).

There are various indications that GLP-1 may regulate the gonadotrophic axis, possibly at the level of the GnRH neurons. Brainstem-derived neural elements directly contact GnRH neurons in male mice (Vastagh *et al.* 2021) and GLP-1 receptors are found in GnRH neurons (Farkas *et al.* 2016). Accordingly, GLP-1 can increase firing of GnRH cells (Vastagh *et al.* 2021). Kisspeptin is a neuropeptide that stimulates GnRH secretion (Ezzat *et al.* 2015), and central GLP-1 neurons also project to kisspeptin cells in mice (Heppner *et al.* 2017). The same authors demonstrated the increased firing of kisspeptin cells with the application of liraglutide, a GLP-1 receptor agonist. Others have reported that GLP-1 increases kisspeptin gene expression in fetal rat brain cultures (Beak *et al.* 1998, Oride 2017), and GLP-1 stimulated GnRH secretion from isolated male rat hypothalami. Intracerebroventricular injection stimulated LH release in rats (Beak *et al.* 1998), which may be due to an effect at the level of the median eminence (ME). Another study in rats (Outeirino-Iglesias *et al.* 2015) presented confusing data, showing that GLP-1 magnified the pre-ovulatory LH surge, whereas exendin-4 (GLP-receptor agonist) paradoxically reduced the surge. In the same study, both agonists had no effect on basal LH secretion in dioestrous rats. GLP-1 had no effect on LH secretion in men (Jeibmann *et al.* 2005).

It has been reported that microinjection of either GLP-1 or exendin-4 into the ME stimulated LH secretion in OVX ewes, strongly suggesting action at the level of GnRH terminals (Arbabi *et al.* 2021). Given that the ME is outside the blood–brain barrier, it is possible that circulating GLP-1 can stimulate the gonadotrophic axis through action at this level. On the other hand, GLP-1 can cross the blood–brain barrier (Fu *et al.* 2020) raising the question as to whether circulating peptide originating from the hindgut acts on kisspeptin and/or GnRH cells. Accordingly, the objective of the present study was to determine the effect of a systemically administered GLP-1 agonist (exendin-4) on LH secretion in ewes. We also confirmed that GLP-1 is produced predominantly in the hindgut of sheep.

## Materials and methods

All *in vivo* procedures were conducted in accordance with the Australian Prevention of Cruelty to Animals Act 1986 and the Australian code of practice for the care and use of animals for scientific purposes. All studies were approved by the Charles Sturt University Animal Care and Ethics Committee, with approval numbers A18029, A19005 and A19385. Ewes of sound health were sourced from the Charles Sturt University teaching flock. The ewes were maintained under climate-controlled conditions with diurnal lighting and provided with food (1.4 kg commercial lucerne chaff mix) twice daily and water *ad libitum*. Ewes were acclimatised to being confined in pens for 1 week prior to the study.

### Experimental procedure for the preliminary study

A preliminary study was performed on 12 5-year-old Merino ewes with a mean body weight of 53 kg (BCS 2.5/5) to ascertain whether the peripheral infusion of the GLP-1 agonist (ab120214; Abcam) stimulates LH secretion during the sheep’s natural breeding season. Ewes were assigned to either follicular phase or luteal phase groups prior to oestrous synchronisation. In the Luteal phase group, oestrus was synchronised by aseptically inserting an intravaginal progesterone-releasing device impregnated with 0.33 g progesterone (EAZI-BREED CIDR, Zoetis Australia, Rhodes, NSW Australia) 21 days prior to the day of sampling. The CIDRs were removed 14 days later and 250IU of equine chorionic gonadotrophin (eCG) (Novormon, Zoetis Australia, Rhodes, NSW, Australia) was administered (i.m.). The synchronisation protocol was the same for the follicular phase group but delayed by 5 days, such that CIDR removal and injection of eCG occurred 24 h before sampling began. The aim of this synchronisation programme was for all ewes in the luteal phase group to have ovulated recently, with an active corpus luteum (CL) and all ewes in the follicular phase group to be in the follicular phase on the day of sampling. The stage of cycle was confirmed with progesterone assays on the morning of sampling.

A 12G catheter (Dwellcath, Tuta Laboratories Australia Pty Ltd, Lane Cove, NSW, Australia) was inserted into the right external jugular on the day prior to sampling. A 75 cm extension set (Global Veterinary Products, Queensland, Australia) primed with heparinised saline (75 units of heparin/mL) and a three-way stopcock attached to allow sampling. A 12G needle was used to insert 0.86 mm medical-grade polyethylene tubing (Sterihealth Laboratory Products Pty Ltd, Dandenong Sth, Vic, Australia) primed with heparinised saline 15 cm into the left jugular vein, which was kept patent with heparinised saline (*vide supra*); this was used for infusions (*vide infra*). A light neck bandage was used to protect both catheters. Patency of the catheter was not maintained in two ewes from the follicular phase group, and these animals were excluded from the experiment, and two ewes subsequently were excluded from the luteal phase group to avoid sample size bias.

A single 5 mL blood sample was collected prior to the sampling period for progesterone assay. Then, blood samples (5 mL) were collected every 10 min for 7 h and two further samples were taken at 30-min intervals for a total sampling time of 8 h. Samples were immediately placed in lithium heparin vacutainer tubes. Packed cell volume was visually assessed throughout the sampling period and remained within normal parameters. The samples were centrifuged and plasma was obtained by aspiration and frozen at −20°C.

After 3 h of sampling, the GLP-1 agonist, 2 mg of exendin-4 was infused for 60 min (i.v.) at a rate of 2 mL/h using syringe pumps (Graseby MS16A syringe driver, Smiths Medical, Minneapolis, MN, USA) attached to the backs of the sheep.

### Experimental procedure for the main study

The experiment was performed in two replicates in the autumn/summer of consecutive years. The ewes in the first cohort were 5 years old with a mean bodyweight of 52 kg (mean BCS 2.5/5). The ewes in the second cohort were 8 years old with a mean bodyweight of 55 kg (mean BCS 2.5/5). Fifteen and 16 ewes, respectively, were recruited into each replicate and underwent oestrous synchronisation, but only 12 ewes from each cohort underwent confinement, treatment and sampling. Acclimatisation and husbandry occurred as described for the preliminary study. Following the results from the preliminary study, all ewes underwent oestrous synchronisation and were treated/sampled in the follicular phase of the cycle. For synchronisation, CIDRs were inserted 13–16 days before the first day of sampling and 125 µg cloprostenol (Estromil, Troy Laboratories Pty Ltd) were administered i.m. 3–6 days before the first day of sampling to cause lysis of any remaining CL. The CIDRs were removed 24 h prior to sampling, causing the onset of a follicular phase of ovarian function (confirmed by measurement of progesterone in plasma).

Indwelling jugular catheters were inserted as described above. Ewes were randomly assigned to three groups: saline (vehicle), 0.5 mg or 2 mg exendin-4, using an online random number generator (*n* = 4 ewes/group). The sampling procedure was as described for the preliminary experiment. Sampling occurred every 10 minutes from *t* = −180 min to *t* = 240 min, relative to the start of infusions. Then, three consecutive samples were taken at 30-min intervals, followed by sampling at 2-h intervals until *t* = 2260 min to monitor the occurrence of pre-ovulatory LH surges. Plasma insulin and glucose assays were performed from samples collected at the midpoint of each experimental period, i.e., before, during and after infusion.

Food was withheld for 12 h prior to the beginning of the sampling period. Following the infusion, each ewe was given an evening and a morning feed throughout the experiment. Behaviour and feed refusals were monitored and recorded.

### Plasma/serum assays

Progesterone levels in serum were measured in a solid-phase, competitive immunoassay using enzyme-labelled chemiluminescent technology (IMMULITE 1000, Siemens Healthcare, Hawthorn East, VIC, Australia).

Plasma LH levels were measured by radioimmunoassays (RIA) (Lee *et al.* 1976) using NIADDK anti-ovine LH-1 as the primary antiserum and NIH-0LH-S18 as a standard. Iodinated ovine LH (^125^I-NIDDK-AFD-9598B) was used as a tracer. Assay sensitivity was 0.1 ng/mL and intra-assay coefficient of variance (CV) less than 10% between 0.337–11.075 ng/mL in the preliminary study. Assay sensitivity was 0.06–0.84 ng/mL and intra-assay CV was less than 10% between 0.407–10.17 ng/mL and inter-assay CV 5.7% at 6.23 ng/mL and 7.9% at 12.29 ng/mL.

Plasma glucose concentrations were measured using an Accu-Chek® Guide Me blood glucose monitor and test strips (Roche Diabetes Care, Mannheim, Germany).

Plasma insulin concentrations were measured in a homologous double antibody RIA. The RIA used purified insulin antiserum raised in guinea pig (Antibodies Australia, Melbourne, VIC, Australia) and purified bovine insulin for iodination and standard (Sigma-Aldrich Pty Ltd, cat#I5500). Assay sensitivity was 0.54 μIU/mL and intra-assay CV 6.9%.

### Preproglucagon gene expression in ewes

This experiment was done to confirm *GCG* expression in the alimentary tract of ewes. Gastrointestinal tissue samples for rtPCR were scavenged from ewes in an experiment at Monash University (Ethics Approval: MARP-2014-054). Ewes were in good body condition with access to food and water *ad libitum* prior to and at time of death and subsequent sample collection. Ewes had undergone oestrous synchronisation using a prostaglandin-based protocol and their stage of oestrous cycle was recorded as either luteal (*n* = 2) or follicular (*n* = 3, 16 h post-prostaglandin injection).

Full-thickness samples of gastrointestinal tissues were collected from the reticulum, rumen, omasum, abomasum, proximal and distal jejunum, proximal and distal ileum, proximal and distal colon and rectum. Samples were washed repeatedly in ice-cold, sterile saline before being snap-frozen in dry ice and stored at −80°C until RNA extraction. The QIAzol Lysis Reagent standard protocol (Qiagen cat#79306) was used to extract RNA from the tissues. Following RNA extraction, cDNA was prepared and genomic DNA removed using QuantiTect Reverse Transcription Kit (Qiagen cat#205310). Real-time PCRs for each sample were set up in triplicate, with 50 ng of cDNA using a QuantiNova SYBR Green Kit (Qiagen cat#208052) on the Rotor-Gene Q real-time PCR machine (95°C 2 min; combined extension and annealing 60°C 1 min for 40 cycles) for all primer pairs. Purified DNA of known concentration was used as the assay standard in each instance. Optimisation of each primer set was achieved by the separation of PCR products by agarose gel electrophoresis followed by purification and sequencing to confirm identity (Table 1). The levels of expression of each mRNA and the estimated concentrations were determined relative to the standard preparation using Qiagen Rotor-Gene Q computer software. Similar amounts of RNA were used for each amplification and the ratio of each mRNA to the geometric mean of three reference genes (cyclophilin, GAPDH and malate dehydrogenase (MDH1)) was calculated to correct for minor differences in the total amount of RNA used between samples.

**Table 1:** Primer sequences for gene of interest and reference genes for measurement of preproglucagon gene (GCG) expression from ovine intestinal samples. Nucleotides in bold are encoded on a separate exon from the remainder of the primer.

Name	Accession number	Forward primer	Reverse primer
GCG	AF529185	**TCCAG**TTCATTCCCAGCTCC	GTGAATGTGCCCTGTGAGTG
GAPDH	MN_001190390	TCAAGAAGGTGGTGAAGCAG	CCCAGCATCGAAGGTAGAA
Cyclophilin	JX534530	GCATACAGGTCCTGGCATCT	CATGCCCTCTTTCACTTTGC
MDH1	XM_004005845	ATCATTCTCGTGCTGTTGGA	CTTCTTTATCCGTGGCGATG

### Statistical analysis

Statistical analysis was performed using R (https://www.r-project.org/). For the purpose of analysis, progesterone values <3 nmol/L were considered consistent with the follicular phase, values >3 nmol/L consistent with the luteal phase.

A multiple linear regression model was used for the analysis of the effect of exendin-4 on LH secretion. Logarithmic transformation of the data was performed after a diagnostic check of the model to achieve homogeneity of variance. Model goodness-of-fit was determined using *R*-squared values and Akaike information criterion (AIC), which suggested a mixed effect model was most suitable.

LH pulse analysis was performed using a previously described method (Clarke 1993) with a pulse defined as having a value higher than three times the assay S.D. of the preceding LH value. Values that fit the definition of a pulse but occurred concurrent with an LH surge were excluded from analysis. Analysis was restricted to the period during which sampling occurred every 10 min. Inter-pulse interval (IPI) was calculated as the interval in time between the start of two adjacent pulses. A mixed-effect regression model was fitted to the data for inferential analysis. A diagnostic check of the assumption conditions for the model suggested a logarithmic transformation was warranted.

An LH surge was defined as a monophasic rise in plasma LH concentration to a value ≥10 ng/mL. The onset of a surge was the first value in a monophasic surge higher than three times the SD of the preceding LH value. Whether treatment had an effect on the time of the onset of surge was assessed using analysis of variance (ANOVA), Kruskal–Wallis and Tukey HSD.

Plasma insulin and glucose concentration analyses were performed using two-way ANOVA and a linear regression model.

Analysis of the RT-PCR results aimed to quantify the association between *GCG* expression using a primer for GCG, site of expression and phase of the oestrous cycle. An ordinary linear regression model was fitted using logarithmically transformed response variables and site and phase of the predictor values. The full factorial model was identified as the optimal model based on AIC.

In all cases, the ‘emmeans’ function of the ‘emmeans’ package in R was used for pairwise comparison (https://CRAN.R-project.org/package=emmeans).

## Results

### Preliminary study

#### Effect of exendin-4 on plasma LH concentrations during the luteal or follicular phases of the oestrous cycle

**Figure 1:**
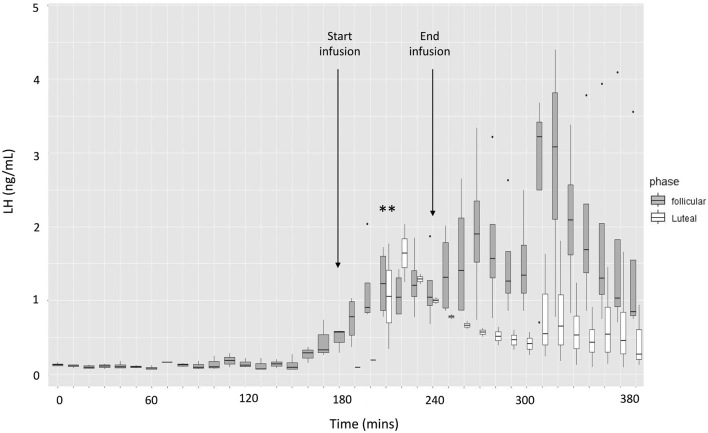
Plasma LH concentrations in the ewes of the preliminary study, treated with 2 mg of exendin-4 in either the follicular (*n* = 4) or luteal phase (*n* = 4) of the oestrous cycle. The data are presented as box plots, where the lower and upper margins of each box represent the 25th and 75th quartiles, respectively. The solid lines within each box represent the median values. The vertical lines represent the minimum and maximum values of the data, and dots represent outliers. The two solid lines mid-graph represent median values in the luteal phase group for which there were too few values to construct a box. ^**^ *P* < 0.01 compared to pre-infusion mean.

Infusion of exendin-4 increased plasma LH (Fig. 1) during and after infusion in both the luteal and follicular phase groups (*P* < 0.001). Mean plasma LH concentrations were higher in the follicular phase group during and after exendin-4 infusion when compared to the luteal phase group (*P* < 0.01 and *P* < 0.001, respectively).

### Main experiment

#### The effect of exendin-4 on plasma LH concentrations in the follicular phase of the oestrous cycle

**Figure 2:**
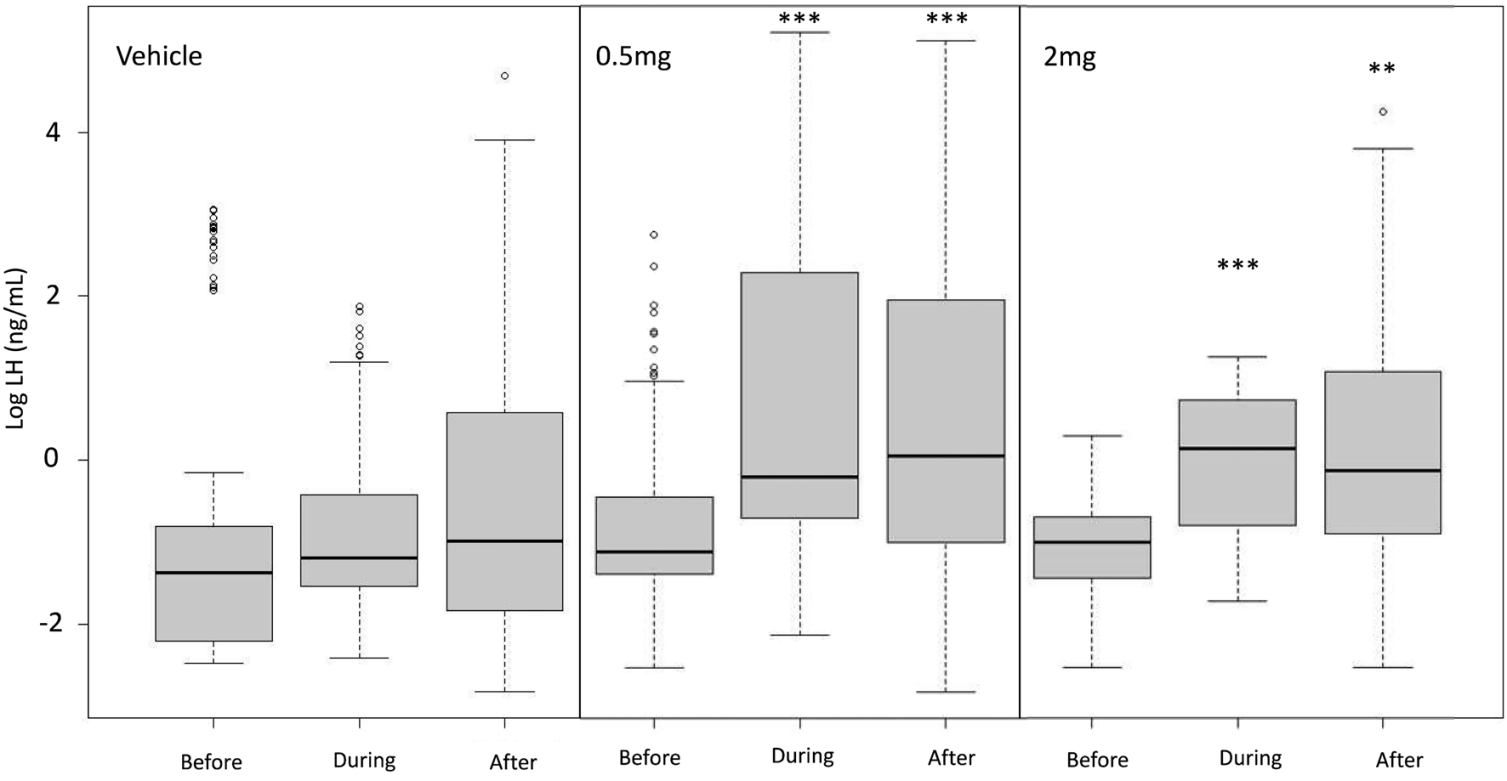
Plasma LH concentrations in ewes in response to either vehicle (control), 0.5 mg or 2 mg of exendin-4. The ewes were in the follicular phase of the oestrous cycle (*n* = 11–12/group). The data are presented as box plots, as described for Fig. 1. ***P < 0.001 **P < 0.01 compared to pre-infusion means.

Two ewes had high progesterone levels during the experimental period (12.6 and 16.9 nmol/L, respectively) and were excluded from all further analysis. A further three ewes experienced an LH surge during the first 3 h of sampling and were also excluded from analysis of pulsatile LH secretion. Exendin-4 infusion increased mean LH concentrations in groups given 0.5 mg and 2 mg of the agonist (*P* < 0.001) (Fig. 2). This effect extended into the post-infusion period in both groups (*P* < 0.001 and *P* < 0.01, respectively) (Fig. 2) although the effect diminished 1300 min post-infusion.

**Figure 3:**
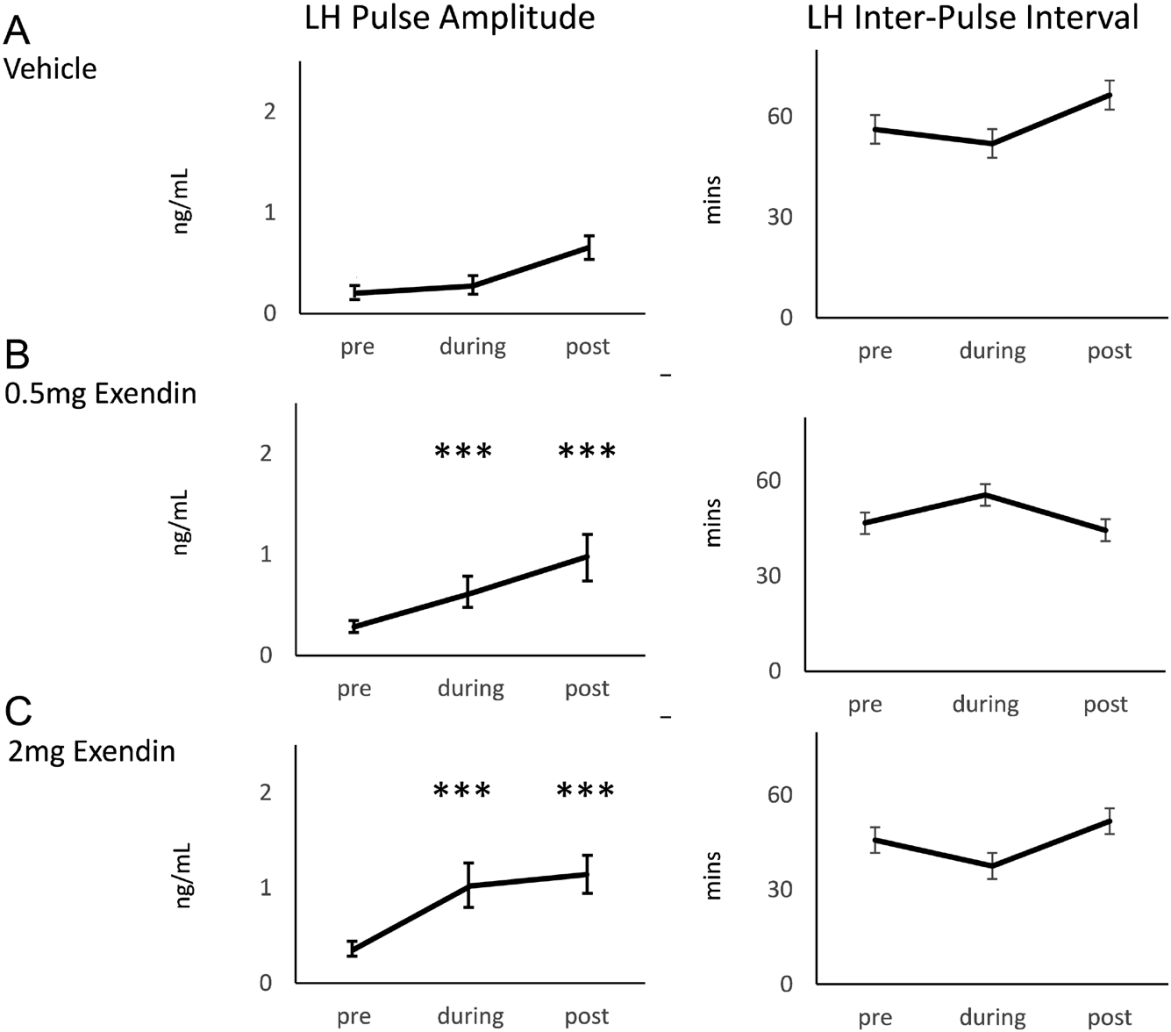
Mean ± s.e.m.values for LH pulse amplitude and LH inter-pulse interval pre-infusion (*t* = −180 to 0 min), during infusion (*t* = 0–60 min) and post-infusion (*t* = 60–240 min) of infusion of exendin-4 in the (A) vehicle (control), (B) 0.5 mg exendin-4 and (C) 2 mg exendin-4 (*n* = 11–12/group). ***P <0.001 vs pre-infusion means

Exendin-4 infusion had no effect on IPI but increased pulse amplitude in both the 0.5 mg and 2 mg treatment groups compared to the saline control (*P* < 0.001) (Fig. 3). The effect on amplitude extended into the post-infusion period (*P* < 0.001). The increase in amplitude was greatest in the 2 mg group (*P* < 0.001), with no significant difference in amplitude between the control and 0.5 mg group and the 0.5 mg and 2 mg groups.

#### The effect of exendin-4 on the onset of the LH surge

**Figure 4:**
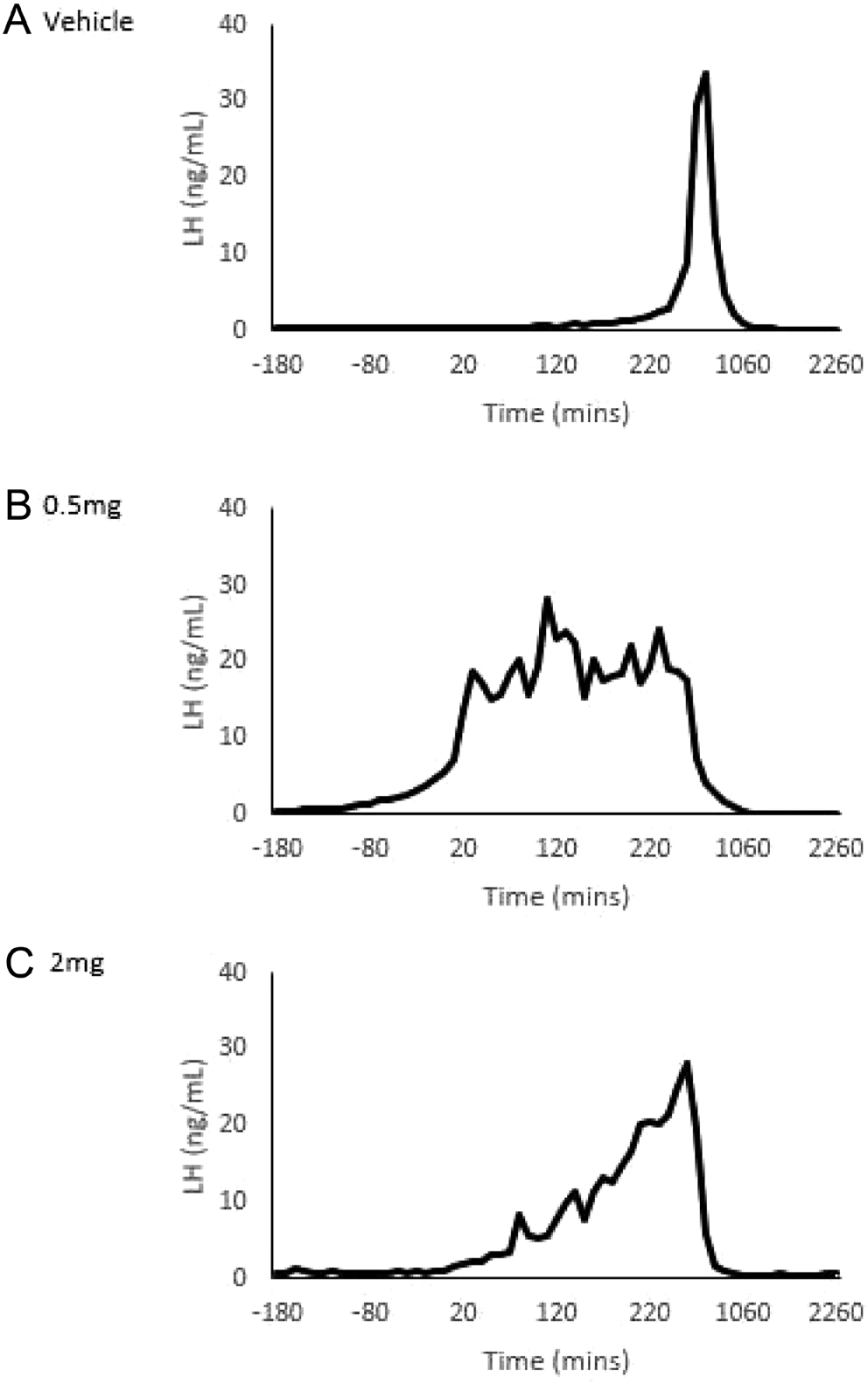
Examples of plasma LH surges in ewes receiving either vehicle (A), 0.5 mg exendin-4 (B) or 2 mg exendin-4 (C), when surges occurred during the time of infusion (beginning at *t* = 0) in the main experiment, with ewe given vehicle for comparison.

Analysis of the onset of LH surge demonstrated that treatment had low predictive power (R-squared = 0.03). Inferential analysis using ANOVA followed by Tukey HSD and Kruskal–Wallis tests confirmed no discernible effect of exendin-4 on the onset of the LH surge. Observation of the data suggests that exendin-4 infusion coincident with the LH surge may have had a modulatory effect, as a protracted surge was seen in ewes in which this occurred (*n* = 2) (Fig. 4).

#### The effect of exendin-4 on plasma insulin and glucose concentrations

**Figure 5:**
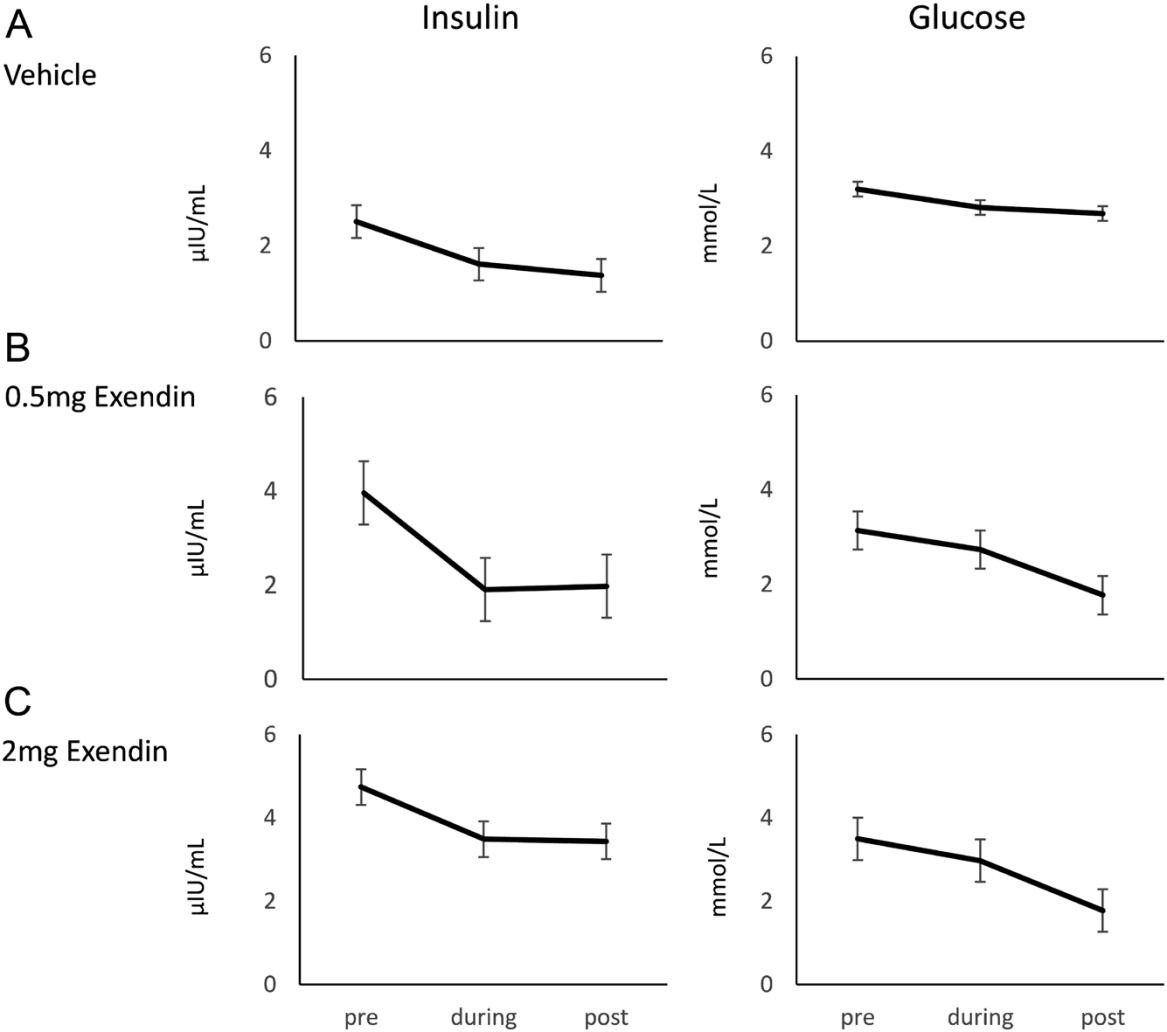
Mean ± s.e.m. concentrations of insulin and glucose in plasma pre-infusion (*t* = −180 to 0 min), during infusion (*t* = 0–60 min) and post-infusion (*t* = 60–240 min) of either vehicle (A) or 0.5 mg exendin-4 (B), or 2 mg exendin-4 (C) (*n* = 11–12/group).

There was no effect of exendin-4 infusion on plasma insulin or glucose concentrations (Fig. 5), but levels of insulin were higher in the 2 mg group than in the control group throughout the experimental period (*P* < 0.05).

#### Expression of the preproglucagon gene in the gastrointestinal tract

**Figure 6:**
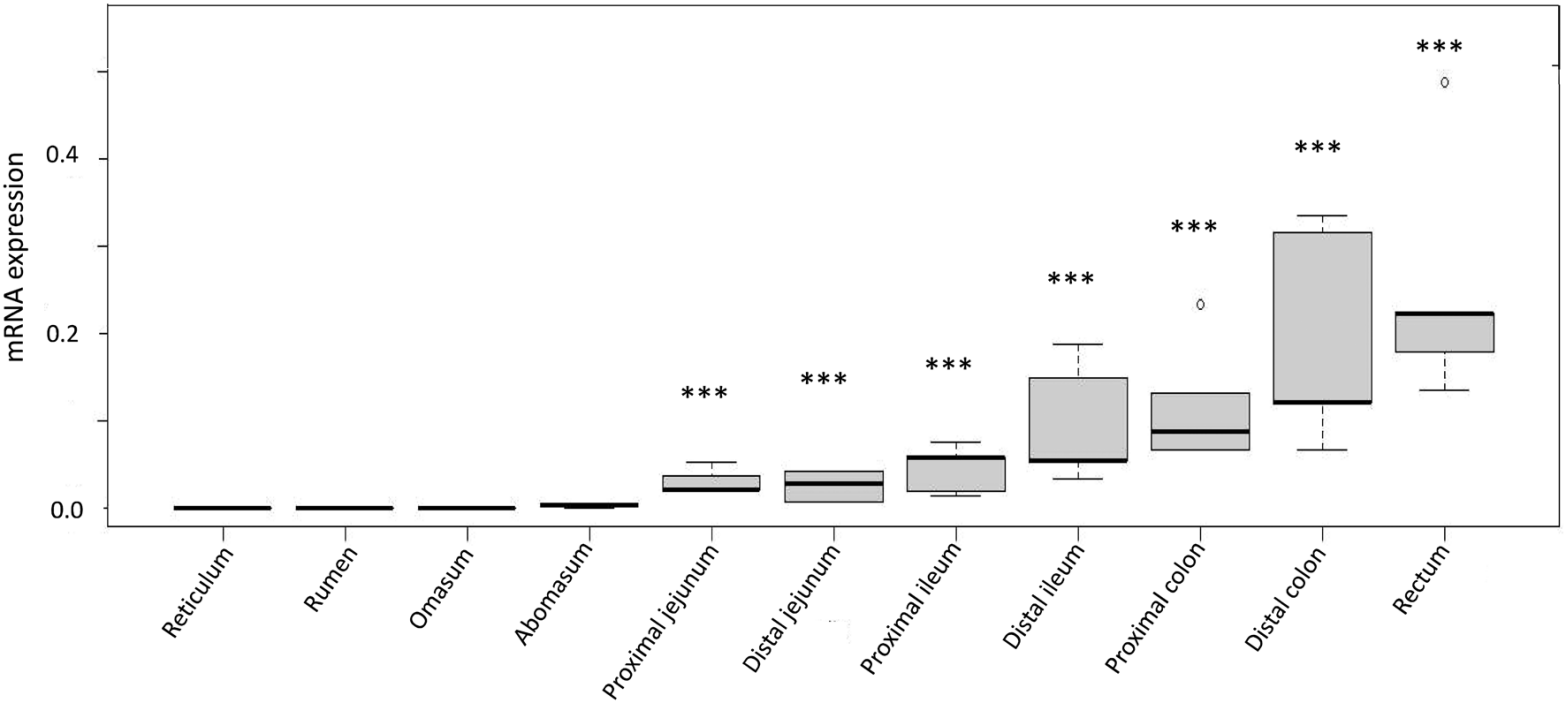
Relative *GCG* expression throughout the gastrointestinal tract of the ewe (*n* = 5). Values for reticulum and proximal jejunum were *n* = 4 and *n* = 3, respectively. The data are presented as box plots, as described for Fig. 1. ***P < 0.001 vs expression in the reticulum.

The level of expression of* GCG* was low in the forestomachs (reticulum, rumen, omasum and abomasum) and increased aborally, with greater (*P* = <0.001) expression in the jejunum, ileum, colon and rectum (Fig. 6). The stage of oestrous cycle had no detectable effect on relative *GCG* expression.

Where they occurred, outliers in the data have been included in statistical modelling.

## Discussion

This study confirms that an agonist of the GLP-1 receptor may stimulate the secretion of LH. We observed that systemic administration of exendin-4 increased secretion of LH in ewes in both the follicular and luteal phase of the oestrous cycle (Fig. 1). This finding is consistent with the general observation that anorexigenic pathways have a stimulatory effect on reproduction (Clarke 2014). An increase in LH secretion occurred with exendin-4 doses of both 0.5 mg and 2 mg, with increased LH secretion typically starting during the infusion period (Figs. 1 and 2). Interestingly, in our study, the effect on LH levels was not sustained into the period following infusion of either 0.5 mg or 2 mg of exendin-4. This may reflect saturation of GLP-1 receptors or negative feedback of the supra-physiological dose of 2 mg on the hypothalamus and/or pituitary, but measurements of both glucose and insulin (*vide infra*) suggest that this was not the case.

Infusion of exendin-4 increased LH pulse amplitude, without effect on inter-pulse interval (Fig. 3). This suggests that the ewe’s inherent GnRH pulse generator was already at its maximal intrinsic rate, as would be expected during the pre-ovulatory, follicular phase. Instead, exendin-4 may have strengthened the signal from the hypothalamus to the pituitary to increase pulse amplitude without affecting the pulse interval. This is supported by our earlier work, which indicated that direct injection into the ME enhanced LH pulse amplitude. This suggests an effect on the release of GnRH at the level of the GnRH neuronal dendrites in the external zone of the ME (Arbabi *et al.* 2021).

We found no effect of exendin-4 on the timing of the preovulatory LH surge. This finding should be considered with caution, given the small sample size and individual variation that was apparent. Alternately, it is possible that GLP-1 may act without affecting the mechanisms generating the GnRH/LH surge, e.g. on GnRH neuronal cell bodies or on pituitary gonadotropes (Clarke 2018, Herbison 2018). Nevertheless, exendin-4 treatment at or near the time of the LH surge may have had a modulatory effect, with a more protracted surge occurring in some treated ewes (*n* = 2) (Fig. 4).

A larger study is warranted to explore this effect. It would be expected, however, that GLP-1 infusion would not amplify the surge, since the pituitary stores of LH are depleted by 90% at the time of the surge in cyclic ewes (Roche *et al.* 1970).

The preliminary study found that hormonal state may influence the effect of GLP-1 or its agonists on LH secretion. Thus, although exendin-4 infusion appeared to result in increased LH secretion in ewes during both the luteal and follicular phases of the cycle, the response was more pronounced and appeared to be more rapid in the ewes in the latter. This suggests sensitivity of the reproductive axis to GLP-1 is greatest during the early oestrous period, when serum progesterone levels are low and oestrogen levels are high. This result is, perhaps, unsurprising, given progesterone exerts a negative feedback effect on GnRH secretion through suppression of kisspeptin drive (Clarke 2014). The ability of exendin-4 to overcome this restriction to some extent in our study is further evidence of the role of GLP-1 in reproduction. Indeed, all the ewes in the luteal group had LH secretion below the assay sensitivity (<0.1 ng/mL) until after the exendin-4 infusion was commenced. The response to GLP-1 at different stages of the oestrous cycle could be due to either (i) increased sensitivity of the reproductive axis to GLP-1 during the follicular phase or (ii) increased expression of GLP-1 receptors in the hypothalamus and/or pituitary during the follicular phase. This could be due to either the increased concentration of oestrogen, low progesterone concentration or a combination thereof that occurs during the follicular phase. Inhibin, IGF-1 or other compounds (e.g. follistatin) could also be involved in the regulation of the response to GLP-1. Outeirino-Iglesias *et al.* (2015) found that GLP-1 receptor expression in the hypothalamus and pituitary varies during the oestrous cycle in the rat, but expression was greatest during dioestrus and lowest during pro-oestrus and oestrus, results which do not explain our observations in sheep and may indicate species differences. The results seen with our use of intact, cycling ewes, predominantly under the influence of oestrogen, compared with those in animals under the influence of progesterone may explain some differences in findings between studies, including the absence of an effect on pulse frequency seen in this study. Our findings suggest ovariectomised animals, male animals and females under the influence of progesterone may have a reduced or altered response to GLP-1 when compared with those under the influence of oestrogen.

We believe this is the first study to demonstrate that systemic administration of a GLP-1 receptor agonist can affect the reproductive axis at the level of the hypothalamus and/or pituitary. Further studies are required to define the level at which this effect is manifest. Both exendin-4 and GLP-1 are able to penetrate the blood−brain barrier (Kastin & Akerstrom 2003) enabling action on kisspeptin and/or GnRH-producing neurons to alter downstream LH secretion. Gut-derived GLP-1 or systemically administered exendin-4 could also act outside the blood–brain barrier at the level of the ME (Arbabi *et al.* 2021) or pituitary gland (Khan *et al.* 2022). The rapid LH response seen to exendin-4 infusion in the present study tends to support the notion that GLP-1 influences GnRH secretion without entering the brain. One ewe appeared to respond during the period the infusion lines were being primed; perhaps due to inadvertent administration of the drug. The ME, which contains GnRH neurons, has a dense population of GLP-1 receptors (Larsen *et al.* 1997) and the increase in LH seen after micro-injection of GLP-1/exendin-4 into the ME suggests that circulating GLP-1 most likely acts on the neurosecretory terminals of GnRH neurons in this area (Arbabi *et al.* 2021). On the other hand, GLP-1 binding to rat pituitary is reported (Shimuzu *et al.* 1987), as is GLP-1R RNA expression (Outeirino-Iglesias *et al.* 2015, Khan *et al.* 2022) and action at this level cannot be excluded. Whether gonadotrophs express GLP-1 receptors is unknown. Other studies of tissue distribution focused on brain and tissues relating to metabolic function (Campos *et al.* 1994, Bullock *et al.* 1996).

The impact of a GLP-1 agonist or GLP-1 is not established with any degree of certainty. The paper of Outeirino-Iglesias *et al.* (2015) presents perplexing data which show that exendin and GLP-1 have opposite effects in rats. These peculiar results require further interrogation. Other papers have reported effects on LH and testosterone secretion in human males.

Jeibmann *et al.* (2005) infused GLP1 (0.4 pmol/kg/min, i.v.) into healthy men, observing no effect on gonadotrophin levels. Jensterle *et al.* (2019) studied men with obesity-related functional hypogonadism, comparing groups treated with either testosterone or liraglutide, finding that the latter showed improvement of gonadotrophin levels. This study did not include a group of men receiving placebo alone. Izzi-Engbeaya *et al.* (2020) infused (i.v.) 0.8 pmol/kg/min GLP-1 or placebo to healthy men and did not observe any effect on reproductive hormones, including gonadotrophins. There are no equivalent studies in women or other species, although GLP-1 receptor agonists are beneficial to the treatment of polycystic ovarian syndrome (PCOS) in terms of weight loss and resumption of menses (Lamos *et al.* 2017, Szeliga *et al.* 2022). Most notable is a study of IVF treatment of infertile, obese PCOS women. In this study, supplementation with liraglutide to metformin treatment raised cumulative pregnancy rates over 12 months from 36% to 69%; gonadotrophin levels were not measured.

Direct micro-injection of either GLP-1 or exendin-4 increased LH pulse amplitude in the OVX ewe (Arbabi *et al.* 2021), strongly suggesting an effect to stimulate GnRH secretion at this level. On the other hand, Farkas *et al.* (2016) found that exendin-4 had a direct excitatory effect on GnRH neurons in brain slices from male mice, but the extent to which centrally produced or circulating moieties may exert an effect is an open question (Farkas *et al.* 2016, Heppner *et al.* 2017, Arbabi *et al.* 2021, Vastagh *et al.* 2021). The GLP1 receptor is expressed in the pituitary gland, ovary and uterus, a finding that suggests GLP-1 may act at multiple levels in the hypothalamic–pituitary–gonadal axis to regulate reproductive function (Outeirino-Iglesias *et al.* 2015, Khan *et al.* 2022). Nevertheless, plasma concentrations of GLP-1 in sheep (Relling *et al.* 2011) and cattle (Zapata *et al.* 2015) are in the picomolar range, so it is realistic to suppose that such levels may be physiologically relevant in terms of control of LH secretion. On the other hand, despite the adoption of the use of GLP-1 agonists for glycaemic control, there are no available data on the impact of such agents on gonadotrophin levels in humans. A recent review of the field (Zhao *et al.* 2021) considered the role that GLP-1 might play in polycystic disease but did not consider its effects on gonadotrophins.

A study examining re-feeding of ewes after a period of feed restriction found restoration of LH pulsatility was associated with increased plasma insulin concentrations. It is possible, therefore, that the incretin effect of GLP-1 is responsible for changes in LH response. Importantly, the lack of response of insulin and glucose to exendin-4 infusion seen in our study demonstrates that the effect of GLP-1 on the reproductive axis is independent of its incretin effect, in ruminants at least.

To confirm that GLP-1 is produced in the gastrointestinal tract of the sheep, we used quantitative PCR and demonstrated that the pattern of *GCG* expression reflects that seen in other species, increasing aborally (Fig. 6; Supplementary Materials, see section on supplementary materials given at the end of this article). Distribution of *GCG* expression in sheep is similar to that in mice and humans, where expression is greatest in the colon and rectum, whilst pigs and rats differ slightly, with expression greatest in the caecum and distal ileum, respectively (Kuhre *et al.* 2014, Paternoster & Falasca 2018). Presumably, GLP-1 is expressed via post-translational processing of *GCG* by intestinal L-cells in sheep, as is reported in other species (Holst 2007, Sandoval & D’Alessio 2015, Paternoster & Falasca 2018). As in other species, the conundrum exists between the distribution of GLP-1-producing intestinal L-cells and the pattern of GLP-1 release, which occurs in a biphasic pattern following eating, consistent with ingesta being present in the stomach and small intestine (Dailey & Moran 2013). It is possible that GLP-1 produced centrally and by the small intestine is responsible for the relatively short-term effects of appetite suppression, incretin effect and the ileal brake. Thus, GLP-1 produced by the large intestine may constitute a baseline secretion responsible for longer-term effects of GLP-1 on reproduction, whilst also moderating glucose metabolism. Accordingly, the products of digestion in the large intestine typically reflect nutrition intake over a few days, rather than a single meal, which could influence the HPG axis. Interestingly, there was some indication that GLP-1 expression may alter with stage of the oestrous cycle, suggesting a reciprocal relationship between the reproductive axis and the gastrointestinal tract, but this requires confirmation with a larger sample size. Expression in the hindgut tended to be higher in the follicular phase, whilst the luteal phase was associated with greater foregut expression, but the small numbers limit statistical interpretation in this study. This may explain some of the differences in findings between our study and Johnson *et al.* (2017), where *GCG* expression was greatest during pro-oestrus in the colon of the rat. That study also found an increase in plasma GLP-1 concentrations during pro-oestrus, suggesting that is when GLP-1 activity is greatest.

In conclusion, we have demonstrated that a GLP-1 agonist can stimulate LH secretion in female sheep when administered systemically. This response varies during the oestrous cycle, being more profound in the follicular phase of the cycle. This suggests that gut-to-brain signaling is a realistic phenomenon, in terms of reproductive function. Our preliminary observation that GLP-1 expression is greatest in the distal regions of the gastrointestinal tract prompts further studies on the expression of *GCG* expression and GLP-1 production in the colon and rectum and possible variation across the oestrous cycle.

## Supplementary Materials

Supplementary Material

## Declaration of interest

The authors declare no conflict of interest which could prejudice the findings of this study.

## Funding

This research was supported by a Charles Sturt University http://dx.doi.org/10.13039/501100001769 Doctorate of Veterinary Studies Scholarship and a research grant from the Graham Centre for Agricultural Innovation. Funding for Open Access publication was provided by the Charles Sturt University http://dx.doi.org/10.13039/501100001769 Open Access Publishing Scheme.

## Author contribution statement

ES, IC, CJS and AG were involved in the design of the study. ES, IC, CJS, CPS and AG contributed to sample collection and experimentation. AR performed hormone assays and PCR laboratory work. ES performed data analysis, and ES, AG, CJS, CPS and IC interpreted the results. ES drafted the paper, and all authors read and finalised the manuscript.
