# Supplementary material for: The GLP-1 agonist, exendin-4, stimulates LH secretion in female sheep
Source: J Endocrinol. 2023 Aug 17;259(1):e230105. doi: 10.1530/JOE-23-0105 (PMC10448581; doi:10.1530/JOE-23-0105)
Supplement: Supplementary Material [file supplementary_material.pdf]

1 acagagctca ggacactgca cacccaaacg agggctcact ctctctccac ctgctctgtt
61 ccacctcctg gtgtcagaag gtaagagtaa tattgagtga ttgtaagatt gtaccttcta
121 tttagggtac agttagttaa gttagggtac atctattcag ttaagtgttc ataaggggc
181 aaagtattat gaattaaagt gctgtttttt caacttatac tttgttatta taatacgtgg
241 ggcagaattg aaatgaaaaa gtgatgtaaa atgcatttaa ataaacgtta acataataaa
301 gcctcaaagc gagtcaaaga aataagtcaa tgaaaagata taagatagaa agaaaaacaa
361 agatgcaaat caaggcactt actacaaaat ttaactaatg aaaattgtat tttaaaaatc
421 agtggtaaca ttttggaaaa cagctatttt tctttggatt cgataaatgt gtatactatt
481 ttgaacagaa ggcgcctttt aaacagcaga aatgtgccca ttaaagaggt tataatagta
541 attgagcctt accaatgaaa acgtatggtt ttccagaaga gcaaaatcaa catcatcatt
601 caaatattca aatttctgat aaggctatca aaaaatcaaa gtccatgaaa tcagctcttt
661 gtagataaga catagcatat ttagggcaga gaagaaatgt catcagttat tgggaatttt
721 gtggatggag ctgagtaaaa gcacagatta ataattacta tgtttggggg accaatatta
781 ataaatagac tatctaaaac gtctacattc caaaatgaaa ttttagtaaa taccttaaga
841 aaaaatatca gatgttctaa agtacctatt taaactctta aggacaagga acaattttac
901 aaagatcttc agtggagaaa atgatatcgt gtttttgtg tttcttggtt ttcacatctc
961 cctgtcaaat tcaggatctt agtttcccaa ccagggatcg aacctgtggc ccctgtattg
1021 ggagcacagt ctctggacca ccagggaagt tccatgtggt atgttttctt tcacacatac
1081 taattaatac ctgtgagaag caattatcta gccagtttta aaagaattta tttggcctat
1141 ggcaccagta tacttaggga tatactatat aatatatggc atcttgccta gatcaaagat
1201 taatctttgc aaaagacttc ctatttagga aatatgattg gtaccacatc tccttttaaa
1261 tttgagtgga aatgtataga ttttattaaa ctgcagactc tctctgtagg ctaaagcagg
1321 ggtgggagag taattggaaa gtctctcctt aatgaataag attttgataa caaaacgatg
1381 caaatgtttg ctttctcaag tagcagtatt aaaaaatcct tatacttaaa tatttcacaa
1441 ttctgttgac ttttacacaa tgaataaata aagtttcaaa aatcaattgt ttatttgaat
1501 agaaatattc taccgcttgg ggaggacttc aaacactgac ttctctatgg ttgtttctta
1561 taccagctct cccagaaact acctgttcca tatctgcctc cagagaatat aatagaagat
1621 attgtaaaac ctttagggac attacatgaa agcaattata gttttcagtt gactttccaa
1681 cagtgatgta atcctaaaga ggaatgtttg ctttaatttt ttatgtttct gaaaatacga
1741 atttataatt aaaaattgca gtcaaagacc ctgcatatat attgggcaag atttccaaag
1801 tttgccaact gtataaactg aagcacagaa acacaccaaa ttagtaattg aatattgata
1861 aataaaggat gaacatgtaa tgcctattgt gttgctttat ctaatcgatc acttaatctt
1921 aaaggcaata tttcaaaggg gaaaaaaaat accactccag gtatttacag tcctcatgtt
1981 ttagtgccaa agaaagatag gaagtatggc taagatgtta aagacaaggt tgatagttta
2041 aaacgataat atttgtaaaa tactcatttc aaaccaaatg ccatttgcta aaaatactca
2101 gtcaaatttt taacaaataa gtataatcaa acttggattg aaatccccat ttctcaaagt
2161 aaatatggtc agacttgaaa gaagcagaga gaagtttaca acaaacactg gatttgaggt
2221 tttcattcag aaaatagagg tgcttgtcct atggggtcgt tctgccccgc actcaggctg
2281 gttccacccc accccatcca cagtacatca tcatgtcctt ggcatcctat ggcctcacgg
2341 ggcatctgct tgtgatgcaa ggtatcttgc aacatggtct agggtgtcac tagcagtgaa
2401 tgacacatcc tggactcagt ccaattataa aacaacctgg ggactttcaa gacttcagaa
2461 gcaagcatta atttcttta tgacagtgat ctcttagttt cttagttgtg attgttcaag
2521 ttgccagaag cattagttct tgttgcaaat tgcttgtctt taaaatgatt tttttccttt
2581 aatttagaac aggtcaaagc accctaaaat acctcaaatc tgttggccgc tttataatta
2641 caaagtctag accaagttac actatttaaa atataaatga ataatacatc tgaaccgaca
2701 gctggtatgc caagttgtag cctgaggctg ctttaagatg gttgaaatgc agtataatgt
2761 aaacgcttgg aagtgaagga ctattaattg aaagccagac tgatagtgaa gagggtggga
2821 tcatggataa ggtgccattc aagctaacca tatgtagcat atcatctaca gtgggtattt
2881 tcaatgttta tcatggacat ttttaatttc aagtctggaa atgagccttc caattattaa
2941 cagaagtgtc ctacacaaat gaagaagttt catagtgcta cacatctttt gtattctttt
3001 tagccaccaa aaaaaaaaaa actgcaagaa aaatataata ttttatatta agctagaaat
3061 ataatcagaa tagaattggt gactagtttt ttctttctac tctcaatatt tacttagtca
3121 aaactgaact aattcagctg acacttgtaa atatggttga gtgtctttat gtctcctttg
3181 actctgtgtt tcaacaggca gcaaaa**atga aaagccttta ctttgtggct ggattgcttg**
3241 taatgctggc acaaggcagc tggcaacatt cccttcagaa cacagaggag aaatccaggt
3301 attaaatctt tagatttgaa ctaacatagc actatgctta ttatacagcc caagataact

3361 gtgactagtc aataggtctc agtaatttgg aataaacacc ctgcacagtt gtcagatgtt
3421 taaagtatca ttgtgataaa ggcttttctg gagtacagat aggagtaggt tatttcataa
3481 ccaagggagt ggggaatgtt gaaaaatatg atacatggga aatgatagct ctttgaaatc
3541 tttggctgat cctgtctgtc cattcattac tcacactgca tggtagatca ttttaattat
3601 tctcaccaac tttttctttt tattttaaaa aataacatca tttcctacta attataaaat
3661 tagtaagaaa ttttacatgt acattcatga tagacaatat gcatgtactg tatgtaaata
3721 catcccagat catttatagc tttgcattgt gtttttctc ttagcatttt attgttagat
3781 tatttttata acattacaaa tatctattaa acgtatgata tttaatgact acatactatt
3841 ccatcttata aatgtacaat atttatttag ctacccgtcc tcttttgaat acttaagtgt
3901 tcttcaaatt ttcctacaaa aagcctgcca ataacatttt tcctacattt cttactattt
3961 tctgaggatt tatttctagt ggtaaaataa ttatgtcaaa ggatttgaat gagtttaaat
4021 tttttataa tatcttgcca acttgctttc cagaaaacta atacacatgc agattcccac
4081 tggcattata gaaattctcc tctcacaata tcctccccgg tatttaatcc ttgctaattt
4141 gacaggtaaa aatagtatct catttctatt ttgagtctta atcatattga atatttcccc
4201 acatagccat gccagccaac tgttgattat tggcatgtgg cttatccacg acatagtttc
4261 caattagcaa gtgggctttt tcttttcttt ctctgaactt tcaattgtct ttccattagc
4321 tgccacatag ccaggtgatg gagctacttt tttatagcaa tatatgcaaa acagaatttg
4381 gaatataaat ctgctgccca aacaaaaatg atttgggacg cttcgctctt ttaaaatatt
4441 caaggcgtgt tgctctcttc tggcattatt aacataatga gtagatgaat ttgttgaaaa
4501 aagatttatt taatcctatt tcagaaaaag agaagccagt tgaaaaatca gttgctagaa
4561 ttccaaatca gagtacattc aactctggta aaaccatggg attcccatgg caaattaagg
4621 tctttctgaa ctgccaacta aaatgctcgg ggaacctgcc ttccagcttc atgtggttcc
4681 atatttgta gagaagctgc tcctcaagtg aatctgctct tgaggagaga tgtagttgca
4741 ccaatcactg ttcttcacag **ttcattccca gctccccaga ccgacccgct cggcgatccg**
4801 **gatcagatca gtgaagacaa gcgccactca cagggcacat tcaccagtga ctacagcaag**
4861 **tacctggact ccaggcgtgc ccaggatttc gtgcagtggc tgatgaatac caagagaaac**
4921 **aa**gtaagaat ttgaacctca tcagaagtgt acgctcaaat gtattactta aatatgtcta
4981 aaattcttcc tgactttctt aagttatcat acctatcaaa taatgcccat aaactcattt
5041 gattctgata atgtttaagc ttggtggtgc taacctccat attatgcctt tgcatgtccc
5101 acccatcacc ccttcaaaac tcttgctgct aagtcgcttc agtcatgtcc gactctgtac
5161 gaccccgtag acggcagccc accaggctct gctgtccttg ggattctcca ggcaagaaca
5221 ctggagtggg ttgccattcc ttctccaatg catgaaagtg aaaaatgaaa gtgaaatcgc
5281 tcagtcgtgt ccgactccta gcgaccccat ggactgcagc ctaccaggct cctccgtcca
5341 tgggattttc caggcaagag tactggagtg ggtgccatt gccttcatta cctaccctca
5401 aatgggacat agactttaaa taattttccc aactttggtc ttagcaatat ttactctgtt
5461 gcccatttta ttttaaaatt gaaagctgac ttttgtgaaa ctgcaaggtt aggaatctaa
5521 ttcctgacca aaagacaaga gcaaattcaa cattctcttg tagggttcaa actcatgact
5581 atgatcacat tcaaaatatc ctctagccaa gttcatcacg gtgagaaatg gaaacagatg
5641 cagtcttttt cttgtagaat acaatccaag atacacatta aaattacagc ctagcagcac
5701 agcataaaga gagaagaagg cacataggaa cacaagccta tcgtaaggca tacattttaa
5761 tgttgaattt ctttgacagt gttggggttt ttgctgcatg aggctactgg aggaaatcag
5821 ccacaagggt ttcacaggga tggtggaatt tcacaatttc tttggagggt ggcccacaaa
5881 gggagggagt tatgcatgca aagtagctga aggaatctaa atatcaggaa aagggaatat
5941 atttgtctgg atagaatgga gaagggtaga gagaaagaga gaaaccagca agaagaatta
6001 aagaggattg ttgaccaaaa attcgtaaag tggagagtac aggaaagatg gtgatcatgg
6061 tcaaagaaga caccagaagt tctagctaag gattacagat ggggcttccc aggtggctca
6121 gtggtattga atccacctac caatgcagga gacataagag atgcaggatc ggtccctgag
6181 tcaggcagat cccctggagg aggaaatggc aacccactct agtattcttg cctggaaaat
6241 tccatggaca gaggaccctg gcgggttaca gtccatgggg tcacaaagag tcggacatga
6301 ctgagctact gagcatgcat tggtgaaaca ggagaaatca caaatttgga aaactgcaaa
6361 ctgtcttcat ttctagagag acaatgtcac aacttggcga atgaacgtaa aataatccta
6421 gctaccaggt ggtttctgtt gtgtttaaca tgaaaccttc cagggcttat atatgatgga
6481 gcagctgacc atctttctgg tcaactcctt ccatggggca ttctgtacac acgtgagggt
6541 gatgtaaggt cattctttta gctttcctca ctgctagatc atcatacatt tcctttaatc
6601 cattatcctg tggtcctttc aatgacattt tgtgacactc ctttgctttc cttcgattca
6661 ataaactaac tctgacccca tgtgttggag taggaaacag aaacccactc cactattcct
6721 gcctggaaaa ttccatggac agtggagcct ggcaggctac agtccatggg gtcccagagt
6781 gagcgacgga gtacgcacac atgaccccag atgtacaaat gacaagctgc tgctttgttt
6841 gcaagtggca tggcacagta gatagccctc acacttttgg actgaagtaa gggcagcttt
6901 aagaattaat gacacagaca agtggaagag gtgaaaatgg gtcactgggt ggagtgaggg
6961 agcttagcct cccttaattg tcgttagatt gcccccatca accttcactc ccaataatta

7021 atgtagcttt ctgatgagtc tcctacacac cctcatctcc ccaaatctcc ctctaagccc
7081 cctccacaca gaatccagaa cccgaagctg ccattgctta taggtgagaa ccatattgct
7141 aaagagacag atcttcaatt taactttcac atttctttca **ggaataacat tgccaaacgt**
7201 catgatgaat ttgagagaca tgctgaaggg acctttacca gtgatgtaag ttcttatttg
7261 gaaggccaag ctgccaagga attcattgct tggctggtga aaggccgagg aaggcgagag
7321 taagtctgta cattcttaat tttttttttt ttttgcttga tgctgaaaac ttagattaca
7381 gttatccata aatggatctg cattatgaag ccattaatat agtctcacaa agtaagggaa
7441 taactcctgt tggtgggaga cagtttataa aagtctcatc tgttttatct ttgagagcaa
7501 gtattaatga tagcatcaca caggttacta tttatcctcc attattagtt gcgttaaaat
7561 taggtattaa tttaccagtc caggatactt ctgaggataa acagagtact tctgataact
7621 acactaggaa aataagctta aattaggact gtccaaaaaa actagggata agggaagaga
7681 agaaataatg ctgaaaactt agtttcattt taaatattca catagtttat gtattaaact
7741 tcattgcagg taaaaatgcg atgaagacag gaaggagcta aagatgttta atgaaatgac
7801 gttcaaaaca aaaatatgtg gaaacattca tctatttaat gatgtatcca tttttttatga
7861 gactaccata gagataatca ttaaattatt acagttgaaa atgagcctaa gacctttagg
7921 tacttccttg gatcttacca cagccaaaca gacccacaga ccaaacagac catctcattt
7981 tcttgtgacc atttgattg actttgaatt atgaaaatca agagattcat gttacactct
8041 atttttatct ttagtggggt tttttttttt tttgccatac cttatggcgt gtgggatcct
8101 agtttcccaa ccatggatca acccaggccc cctgaagttg gaaacacaga gtattaacca
8161 ctggaccatc agggatgtct ttagtgagat tttcaacaag agtattgaga ttttaaggag
8221 ctctcaagac aaaagggaac tagtattgtc actgattttt aaaacagcag caataaaatt
8281 tataaaatca atgggaaaat agtcacacaa gattatttta tagtgtttta aagagatacc
8341 ccaattctgt taaacaaacc agagaaaata tctaatatag actaaggttt aatcccttaa
8401 gactgtactt ggcaaagctg ttttgctcat cattagtttt ataggatata ttatattttt
8461 ctgaatatgc tgctgctgct gctgctaagt tccttcagtc gtgtccgact ctgtgtgacc
8521 ccagagacgg cagcccacca ggctcccccca accctgggat tctccaggca agaacactgg
8581 agtgggttgc catttccttc tccattatct gaatacagtc aaatcaatag tttcaataat
8641 aagttttatg ttgacaatac actaaaatct tagaagcagc tggaatacag aaaagggctt
8701 tgtttcacgt cctggctctg cgcttgtcta cctaattcgc tttctgacat tcaagttatg
8761 aggcttttttg tggtaagccc tctgtaaata gaaacatatg gtttctcagt gacccttaca
8821 caggttacca ggagcaggca accaataagc atttctttga aatgtttaac caaatgctga
8881 ttttacccct ccatttctca **gtttcccgga agaagtcaac atcgttgaag aactccgccg**
8941 cagacacgcc gatggctctt tctctgatga gatgaatacc gttctcgata gtcttgctac
9001 ccgagacttt ataaactggt tgcttcagac gaaaattact gacaggtgac tgtcttttgg
9061 ttcatccctg aaaccatcaa aactttcata ggtattatca tactgctata ggctgttgct
9121 acatgaagga aagagtcttt actctcttgg tatacaatca aaattatgaa tcattcaaga
9181 gtatattatt atatttgttt ctgatcatta caattttatc ctttcactca ttgattccaa
9241 atacttttcc tgaatgttgg taagggacaa gttcaaaaca caaacggatg cataaaagga
9301 gaagatgaac agtctttatc aaaataaatc atcgaaacat tttctggcta agttaactgt
9361 atcatctatg tattatacta tgttaaataa tcattgctct ctttttttttt aatttctag**g**
9421 **aagtaa**gtgt gtcattcatt actcaagatc atcttcacaa tatcacctgc cagccatgtg
9481 ggatgtttaa aattttaagt tctgtaaatt taacagctgt attctaaagc catattgctt
9541 gcatgcaaat aaataaattt ccttttaata ttgtataacc aaaagattat aaattgaata
9601 caccattgtc aaaatagtgc taaaatatca gctttaaaat atgataattc agaattctat
9661 ttcttttctt ctgctaatct gcatagcaat gaaattattt ctctgtgata taatttgtat
9721 atataaatta ctccaatcac aacatatttg cattctaata agataagggg gaggactggt
9781 agccacagtt gtgagatggg aaagagaatt ttcttcttga aacttttgtc ataaaaatgc
9841 tcagctttca gtatataaaa gataaactaa ataaaaattt caagcttctt ca
